# Complete Genome Sequence of *Thermoanaerobacterium* sp. Strain RBIITD, a Butyrate- and Butanol-Producing Thermophile

**DOI:** 10.1128/genomeA.01411-17

**Published:** 2018-01-11

**Authors:** Ranjita Biswas, Marcel Huntemann, Alicia Clum, Manoj Pillay, Krishnaveni Palaniappan, Neha Varghese, Natalia Mikhailova, Dimitrios Stamatis, T. B. K. Reddy, Chris Daum, Nicole Shapiro, Natalia Ivanova, Nikos C. Kyrpides, Tanja Woyke, Adam M. Guss

**Affiliations:** a Centre for Rural Development and Technology, Indian Institute of Technology Delhi, Hauz Khas, New Delhi, India; b DOE Joint Genome Institute, Walnut Creek, California, USA; c Biosciences Division, Oak Ridge National Laboratory, Oak Ridge, Tennessee, USA

## Abstract

*Thermoanaerobacterium* sp. strain RBIITD was isolated from contaminated rich growth medium at 55°C in an anaerobic chamber. It primarily produces butyrate as a fermentation product from plant biomass-derived sugars. The whole-genome sequence of the strain is 3.4 Mbp, with 3,444 genes and 32.48% GC content.

## GENOME ANNOUNCEMENT

*Thermoanaerobacterium* sp. strain RBIITD was isolated from a contaminated rich growth medium in an anaerobic chamber. It is a thermophilic anaerobic rod-shaped member of the *Firmicutes* that ferments various plant biomass-derived sugars, including glucose, xylose, arabinose, maltose, fructose, cellobiose, galactose, lactose, mannose, maltose, rhamnose, and sucrose, primarily into butyrate, with the additional production of lactate, acetate, H_2_, and *n*-butanol, with no detectable ethanol and acetone production. The strain is interesting from an industrial standpoint due to its exceptionally high yield of butyrate from xylose and glucose (approximately 85% and 60% of the theoretical maximum yield, respectively). Butyrate is a 4-carbon organic acid that is primarily petroleum derived, but bio-based processes are in high demand for applications in the food/feed industry; as a biofuel or jet fuel precursor; in the cosmetic, plastic, and textile fiber industries; and as a bioactive compound in the nutraceutical industry (1–3). This strain could help fill the gap between the demand for bio-based butyric acid and the lack of availability of natural microbes to produce butyric acid on a large scale from plant sugars.

The draft genome of *Thermoanaerobacterium* sp. RBIITD was generated at the DOE Joint Genome Institute (JGI) using the Paciﬁc Biosciences (PacBio) sequencing technology (4). A PacBio SMRTbell library was constructed and sequenced on the PacBio RS platform, which generated 176,912 ﬁltered subreads totaling 555.0 Mbp. All general aspects of library construction and sequencing performed at the JGI can be found online (http://www.jgi.doe.gov). The raw reads were assembled using HGAP version 2.2.0.p1 (5). The ﬁnal assembly contained 1 contig in 1 scaffold, totaling 3.4 Mbp. The input read coverage was 164.7×.

Genome annotation was performed using the DOE-JGI annotation pipeline (6, 7). Genes were identiﬁed using Prodigal (8), followed by a round of manual curation using GenePRIMP (9). The predicted coding sequences (CDSs) were translated and used to search the Integrated Microbial Genomes (IMG) nonredundant database and the UniProt, TIGRFam, Pfam, KEGG, COG, and InterPro databases. The tRNAscan-SE tool (10) was used to ﬁnd tRNA genes, whereas rRNA genes were found by searches against models of the rRNA genes built from SILVA (11). Other noncoding RNAs, such as the RNA components of the protein secretion complex and the RNaseP, were identiﬁed by searching the genome for the corresponding Rfam proﬁles using Infernal (12). Additional gene prediction analysis and manual functional annotation were performed within the Integrated Microbial Genomes (IMG) platform (13) developed by JGI (14).

The genome sequence length is 3,402,993 bp, with 32.48% GC content. The total number of predicted genes is 3,444, of which 3,348 are protein-coding genes, and 2,576 genes had a functional prediction. A total of 96 RNA genes were determined, including 5 rRNA operons. The whole-genome sequence of this strain will offer insight into its metabolic network, serve as a new source for thermophilic proteins, and provide necessary information to enable metabolic engineering for the production of renewable fuels and chemicals from plant biomass feedstocks.

### Accession number(s).

The complete genome sequence of *Thermoanaerobacterium* sp. strain RBIITD has been deposited in GenBank under the accession number LT906662.
